# When can nonveridical preferential attitude predicates take questions?

**DOI:** 10.1007/s11049-026-09729-0

**Published:** 2026-10-20

**Authors:** Ciyang Qing, Deniz Özyıldız, Floris Roelofsen, Maribel Romero, Wataru Uegaki

**Affiliations:** 1 Linguistics and English Language, School of Philosophy, Psychology and Language Sciences, University of Edinburgh, Dugald Stewart Building, 3 Charles Street, Edinburgh, EH8 9AD UK; 2 Institute for Linguistics, University of Stuttgart, Keplerstr. 17, D-70174 Stuttgart, Germany; 3 Institute for Logic, Language and Computation (ILLC), University of Amsterdam, P.O. Box 94242, 1090 GE Amsterdam, The Netherlands; 4 Department of Linguistics, Room G222, Fach 181, Universitaetsstr. 10, University of Konstanz, D-78464 Konstanz, Germany

**Keywords:** Clause embedding, Attitude predicates, Selectional restrictions, Veridicality, Preferentiality, Clausal distributivity

## Abstract

A growing body of evidence suggests that whether or not attitude predicates may combine with question complements is determined at least in part by some of their semantic properties. Zooming in, nonveridical preferential predicates (NVPs) such as *hope* and *fear* have been claimed not to combine with questions, but this empirical generalization as well as its proposed explanation have been challenged by a number of counterexamples, whose properties—beyond the fact that they have the predicate combining with embedded questions—remain ill understood. By taking a closer look at the combinatorial and semantic properties of different NVPs from English, Japanese, Mandarin Chinese, Spanish, and Turkish, this work narrows down the semantic properties of attitude predicates that determine their combinatorial properties in a crosslinguistically informed and predictive way. We propose a new classification of NVPs based on two empirical properties: C(lausal)-distributivity and valence, and show that non-C-distributive NVPs (e.g., *worry*) as well as evaluatively negative and C-distributive NVPs (e.g., *fear*) can in fact combine with questions.

## Introduction

One of the long-standing questions in the semantics of attitude predicates concerns whether the combinatorial (or selectional) restrictions of attitude predicates are related to their lexical semantic properties, and if so, how. Much of the literature has engaged with a particular instance of this question, namely with the distinction between so-called responsive predicates, i.e., predicates that can take both declarative and interrogative clauses, and antirogative predicates, i.e., predicates that can take declarative but not interrogative clauses (Zuber 1982; White and Rawlins 2018; Theiler et al. 2019; Uegaki and Sudo 2019; Özyıldız 2021; Roberts 2021; Djärv 2023).^1^ In (1) are examples of responsive (1-a) and antirogative (1-b) predicates:

**Figure:**
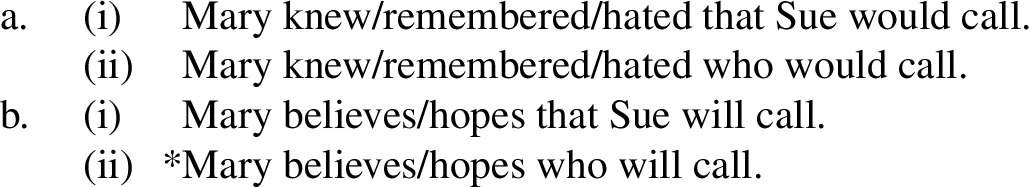

These authors observe that these predicates’ combinatorial restrictions correlate with and could be explained by semantic properties like veridicality, neg-raising, preferentiality or eventivity. Theiler et al. (2019), Mayr (2019), and Uegaki and Sudo (2019) provide concrete accounts for some classes of antirogative predicates, based on the idea that the combination of certain semantic properties with interrogative complements results in logical triviality, which is then assumed to lead to unacceptability (following Gajewski (2002) and others’ proposals about meaning-driven unacceptability). More specifically, Theiler et al. (2019) and Mayr (2019) focus on an empirical generalization stating that neg-raising predicates are antirogative (Zuber 1982), and explain this generalization based on the idea that when a neg-raising predicate is composed with interrogative complements, the Excluded Middle property (argued originally by Bartsch 1973 to derive the neg-raising inference) results in semantic triviality. Building on this enterprise, Uegaki and Sudo (2019) suggest that nonveridical preferential predicates (NVPs) are antirogative as well, and provide an account of this descriptive generalization based on the triviality that arises from the combination between interrogative complements and the conjunction of nonveridicality and preferentiality. The definition of these semantic properties will be discussed in Sect. 2.1.

Although these accounts provide interesting analytical possibilities and serve as important stepping stones to addressing the overarching question on combinatorial restrictions, issues still remain with respect to the empirical status of the purported correlations between combinatorial restrictions and lexical semantics. Indeed, White (2021) presents attested counterexamples to both of the generalizations noted above, i.e., that neg-raising predicates are antirogative and that nonveridical preferential predicates are antirogative. Furthermore, data considered in the current literature mostly come from English, while the crosslinguistic status of the relevant generalizations have not been systematically investigated. This is a crucial gap in the current empirical landscape, as the theoretical accounts of the correlations between combinatorial and semantic properties cited above predict that the same correlations should hold crosslinguistically because they rely on general compositional-semantic mechanisms which are not assumed to be language-specific.

The primary goal of this paper is thus to improve our understanding of the crosslinguistic empirical landscape concerning the correlations between attitude predicates’ semantic and combinatorial properties. Our focus will be on nonveridical preferential predicates, such as *hope*, *fear*, and *worry* in English as well as some of their crosslinguistic counterparts. Our point of departure is Uegaki and Sudo’s (2019) analysis of NVPs that derives the generalization that they are antirogative. We outline their assumptions in Sect. 2.1. (Our focus on this proposal is due to the fact that it is the only fully fleshed out one on NVPs, and in particular, on their purported antirogativity. We leave the exploration of alternative proposals for another occasion.) In the following sections, we will provide counterexamples (some original and some from the literature) to Uegaki and Sudo’s (2019) generalization from English, as well as from Japanese, Mandarin Chinese, Spanish, and Turkish. These counterexamples show that Uegaki and Sudo’s (2019) descriptive generalization is not tenable in its original form, but they also reveal fine-grained variations in the lexical semantics of NVPs, which, to our knowledge, have not been documented in the previous literature.

To preview, we will show that NVPs can be divided into three classes, varying across two factors: (i) *C(lausal)-distributivity* (i.e., the validity of the following biconditional for any exhaustivity-neutral question *Q*: ⌜*x Vs Q*⌝ iff there is an answer *p* to *Q* such that ⌜*x* *Vs* that *p*⌝; see Spector and Egré 2015, Theiler et al. 2018) and (ii) *valence*, i.e., whether the predicate is evaluatively positive or negative. Among the three classes, only one class, i.e., C-distributive and positive NVPs, seems to exhibit antirogativity (at least under classical assumptions about composition with embedded questions to be discussed below), while the other two classes—[non-C-distributive] and [C-distributive, negative]—are both compatible with interrogative complements. This empirical picture is summarized in Table 1.

**Table 1:** Summary of the selectional behavior of NVPs depending on C-distributivity and valence, i.e., evaluative positivity/negativity, with section numbers for relevant discussion

	non-C-distributive	C-distributive
positive	✓*V Q*, Sect. 3.1.1	*V Q (under classical composition)
negative	✓*V Q*, Sect. 3.1.2	✓*V Q*, Sect. 3.2

In the rest of the paper, we will argue that examining the assumptions in Uegaki and Sudo’s (2019) formal analysis lets us clarify what their formal analysis actually predicts and illustrate that it in fact provides theoretical explanations for these empirical observations. Specifically, in Sect. 2, we will review the derivation of triviality for NVP + *Q* in Uegaki and Sudo’s analysis and highlight its two crucial assumptions: (i) NVPs are C-distributive and (ii) NVPs have a Threshold Significance Presupposition, i.e., the presupposition, of a sentence like ⌜*x* *Vs* *Q*⌝, that there is a proposition in *Q* that exceeds the threshold determined by the context and the choice of *V*. We show in Sect. 3 that neither assumption holds in general. We first provide examples of non-C-distributive NVPs in Sect. 3.1. Since such predicates do not meet Uegaki and Sudo’s assumption (i), their compatibility with interrogative complements is straightforwardly expected. Furthermore, we show in Sect. 3.2 that evaluatively negative predicates systematically lack the Threshold Significance Presupposition, explaining the compatibility of [C-distributive, negative] NVPs with interrogative complements.^2^ Along the way, we will provide data from English, Japanese, Mandarin Chinese, Spanish, and Turkish that are consistent with the classification in Table 1. The main goal is to provide support for existential claims about the three classes and their combinatorial patterns. That is, we provide concrete attested examples of classes that are predicted to be possible (i.e., *V* + *Q* for [non-C-distributive] and [C-distributive, negative] NVPs). Our crosslinguistic investigation, despite being relatively small-scale, improves our understanding of the combinatorial patterns and restrictions of NVPs in ways that would be hard to achieve if we only consider data from English (or any single language), as there may be accidental and idiosyncratic factors that make predicates of a certain class hard to find or even nonexistent in a single language. For instance, while evaluatively positive non-C-distributive predicates are hard to find in English (or Spanish and Turkish), attested examples are more readily available in Mandarin and Japanese. Section 4 concludes.

## The baseline generalization about nonveridical preferentials

### Uegaki & Sudo’s (2019) descriptive generalization and formal derivation

Based on examples like (2), Uegaki and Sudo (2019) (U&S) conjecture that nonveridical preferential attitude predicates are antirogative, i.e., that they cannot take interrogative clauses, while being able to take declarative ones. This class of predicates includes *hope*, *wish*, *expect*, and *fear*, as shown, but also *want*, *be eager*, *aspire*, *desire*, or *prefer*.

(2)**Figure:**
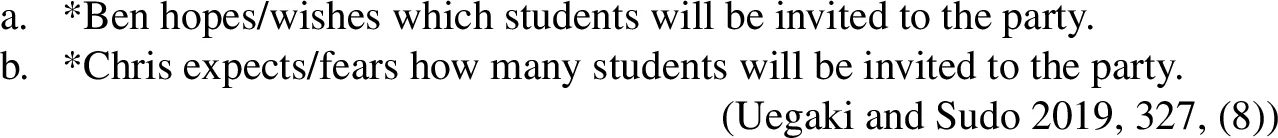

In contrast to their nonveridical counterparts, *veridical* preferential predicates—often also known as emotive factives—are responsive, i.e., they are compatible both with declarative and with interrogative clauses. Their compatibility with interrogatives is shown in (3). This class includes, but is not limited to, *be surprised*, *be annoyed*, *be glad*, *be happy*, *like*, *love*, and *hate*. We note, without attempting to say why, that some of these predicates sound better with embedded questions when these are introduced by a preposition. Prepositions that introduce questions are discussed by Mayr (2019), Egré (2008: Sect. 4.3), Rawlins (2013), and Uegaki and Sudo (2019: Sect. 5).

(3)**Figure:**
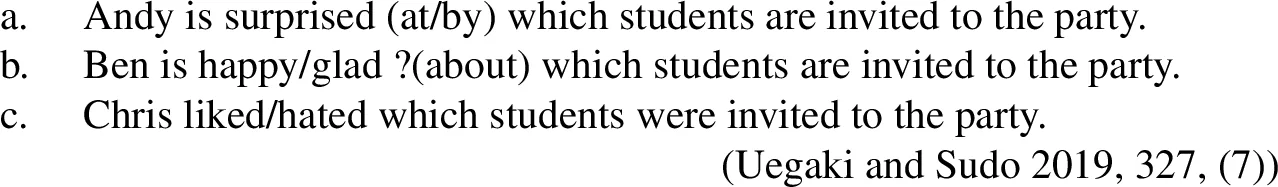

A word about what makes a predicate a (non)veridical preferential: For a predicate *V* to be veridical, it is necessary and sufficient that sentences of the form ⌜*x* *Vs* *that* *p*⌝ or ⌜*x* *Ved* *that* *p*⌝ entail *p*. For a predicate *V* to be preferential, U&S hypothesize that a sufficient condition is for *V* to be focus-sensitive and gradable (Villalta 2008, Romero 2015, a.o.). Focus-sensitivity means that differences in the position of focus within *V*s *that*-clause complement give rise to differences in the truth-conditions of ⌜*x* *Vs*/*Ved* *that* *p*⌝. And gradability means that *V* can be modified by degree adverbials (*x is very surprised that p*) and participate in comparative constructions (*x is more surprised that p than y is*). Both of these properties factor into the lexical entries from U&S provided below, with the reference to a set of alternative propositions *C* to preferential predicates’ complement (focus-sensitivity), and to a comparison between degrees in the predicates’ assertion (gradability).

U&S then propose a formal derivation of the (ostensible) incompatibility between nonveridical preferentials and interrogative complements. Note that U&S limit themselves to attitude constructions that involve *classical composition*, according to which (i) the attitude predicate maintains its standard meaning (e.g., there is no event coercion), (ii) the polar question has a bipolar denotation (of the form {*p*,¬*p*}) and (iii) this question denotation serves as the semantic argument of the predicate. Their account rests on three general premises. First, embedded declaratives and interrogatives both denote objects of the same type, and attitude predicates are not restricted by their lexical entries for whether or not they are able to combine with one or the other clause type (Uegaki 2015; Theiler et al. 2018). In the case at hand, both veridical and nonveridical preferentials will compose freely with declarative and with interrogative complements. A consequence of this treatment is that it is meaningful to compute and reason about the semantic values of (certain) sentences that are ungrammatical, like the ones in (2).

Second, as will be further illustrated in this section, semantically composing certain attitude predicates with certain clause-type denotations gives rise to meanings that are logically trivial—tautologies or contradictions—in a systematic way. We do not fully elucidate what ‘a systematic way’ means here, but U&S understand it as Gajewski’s (2002) notion of L-analycity: A sentence has an L-analytical meaning if its meaning is tautologous or contradictory regardless of which nonlogical lexical items are used to construct sentences with the same structure. In U&S’s account, the result of combining nonveridical preferentials with interrogative denotations, given a reasonable hypothesis about their lexical semantics, is systematically tautologous. In contrast, composing nonveridical preferentials with declaratives and veridical preferentials with declaratives or with interrogatives avoids any logical triviality.

Finally, the grammar filters out as ungrammatical sentences that are assigned certain logically trivial meanings, in particular L-analytical ones (Barwise and Cooper 1981; Chierchia 2013; Gajewski 2002). Conjoined with the second premise, the result is that nonveridical preferentials give rise to ungrammaticality when they compose with interrogatives. In contrast, nonveridical preferentials with declaratives and veridical preferentials with declaratives or interrogatives are not expected to give rise to ungrammaticality.

We now elaborate on the second premise. Here, we present a simplified version of U&S’s formal analysis of preferential predicates, focusing on aspects that are central to their derivation of the incompatibility between nonveridical preferential predicates and interrogative complements. In particular, we will highlight two crucial assumptions U&S made about preferential predicates (veridical or not).

U&S propose the following degree-based semantics for the at-issue content of nonveridical preferential predicates taking declarative clauses (4).

(4)**Figure:**
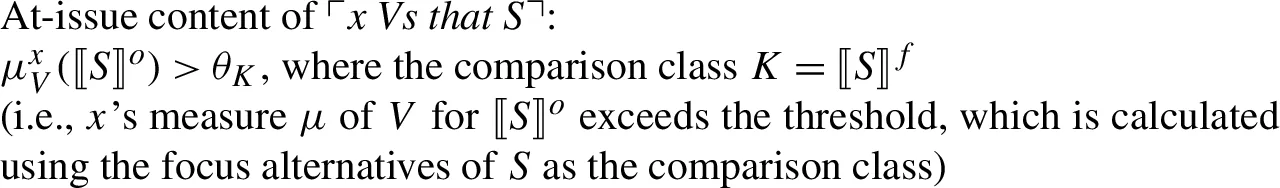

For instance, the at-issue content of *John hopes that MARY*_*F*_ *will sing* is in (5).

(5)**Figure:**
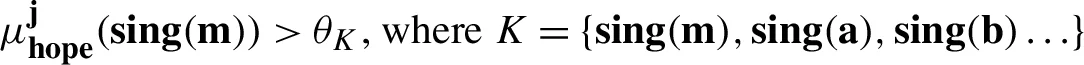

When it comes to embedded questions, given that (6) is ungrammatical, we do not have direct access to its meaning. However, from the perspective of parsimony, it is reasonable to assume that nonveridical and veridical preferential predicates are only different in terms of veridicality, which means that one may infer what the semantics of nonveridical preferentials taking questions should be, from the semantics of veridical preferentials taking questions. Specifically, U&S make two critical assumptions about the semantics of (6), which can be motivated based on the semantics of (7).

(6)**Figure:**
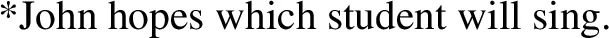

(7)**Figure:**
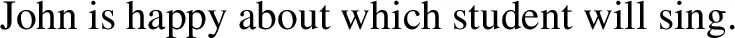

The first assumption is *clausal distributivity*, or *C-distributivity* for short (Theiler et al. 2018). A predicate *V* is *C-distributive* iff the following (8) holds for any *x* and any exhaustivity neutral *Q*. (An interrogative complement is *exhaustivity neutral* iff its interpretations at various exhausitivity levels, i.e., strongly exhaustive, intermediate exhaustive, and mention some, all coincide. Most notably, polar and singular-*which* questions are exhaustivity neutral.)

(8)**Figure:**
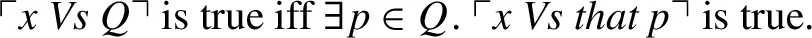

Most predicates (whether veridical or not) are C-distributive (9), including in particular the veridical preferential predicate *be happy* (10).

(9)**Figure:**
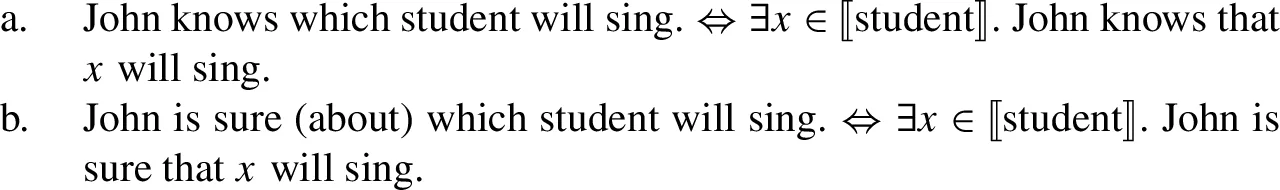

(10)**Figure:**
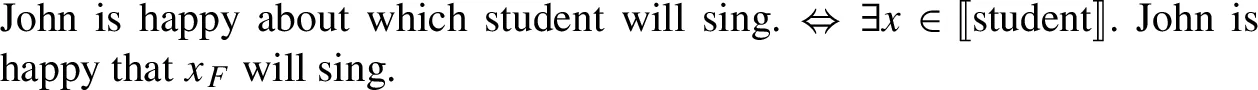

Therefore, it seems reasonable as a first pass to assume that *hope* is also C-distributive (11), from which we obtain the at-issue content of (6) in (12).

(11)**Figure:**
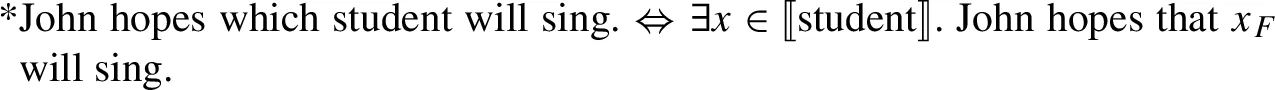

(12)**Figure:**
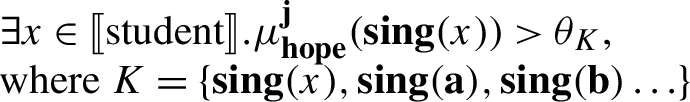

U&S’s second assumption regarding the meaning of (6) is that it carries a what they call *Threshold Significance Presupposition*, according to which the interrogative complement has a possible answer whose relevant measure (as specified by the attitude predicate) exceeds the contextual threshold (13).

(13)**Figure:**
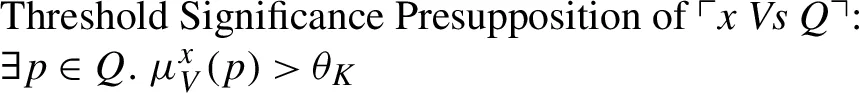

Their empirical motivation comes from the following observation about *be happy*.

(14)**Figure:**
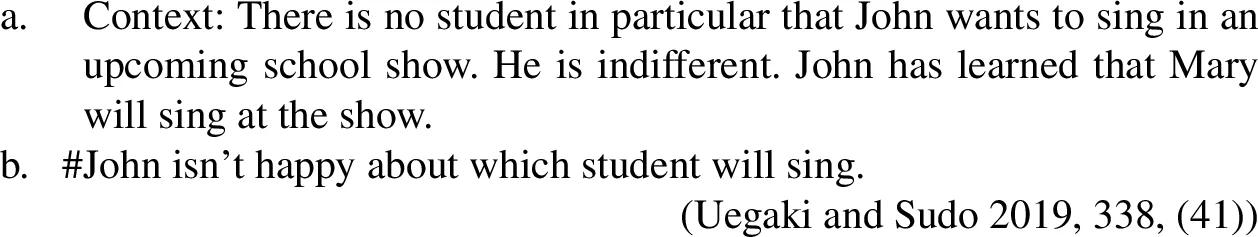

According to (10), (14b) is true in (14a), but the sentence nevertheless feels infelicitous in this context. Intuitively, (14b) implies that John has a preference, which contradicts the contextual assumption that John is indifferent. This intuition is captured by the Threshold Significance Presupposition (13), according to which there exists a student *y* such that if *y* will sing, John will be happy to an extent that exceeds the contextual threshold, and therefore John is not indifferent.^3^

Under U&S’s two assumptions, (6) is predicted to have the meaning in (15).

(15)**Figure:**
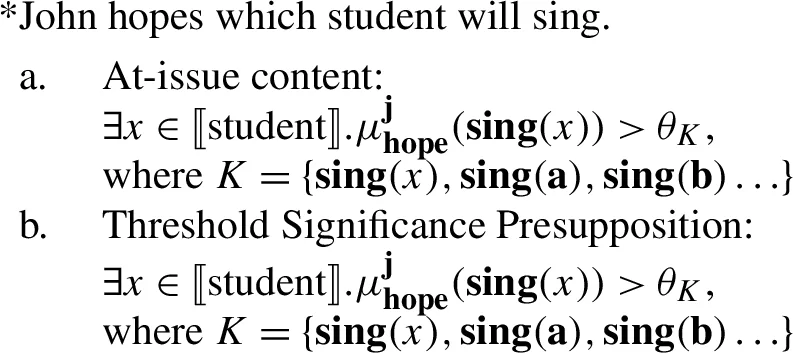

Crucially, this sentence’s at-issue content is the same as its Threshold Significance Presupposition. That is, the sentence is semantically trivial as it does not convey anything beyond what it already presupposes. Moreover, this semantic triviality is systematic as it holds for any interrogative complement. Therefore, U&S take this to account for the ungrammaticality of *hope* (and nonveridical preferential predicates in general) taking interrogative complements.

### Some empirical challenges to Uegaki and Sudo’s original descriptive claim

There have been empirical challenges to U&S’s original descriptive generalization that nonveridical preferentials are antirogative.

Uegaki (2022) reports on an experimental study where participants were presented with a set of 87 preferential predicates and rated their veridicality and compatibility with interrogative complements using a 7-point Likert scale. The experiment was done in two blocks: the veridicality block and the compatibility-with-interrogatives block. See Uegaki (2022) for details of the methodology including the procedure according to which the predicates included were selected.

**Fig. 1:**
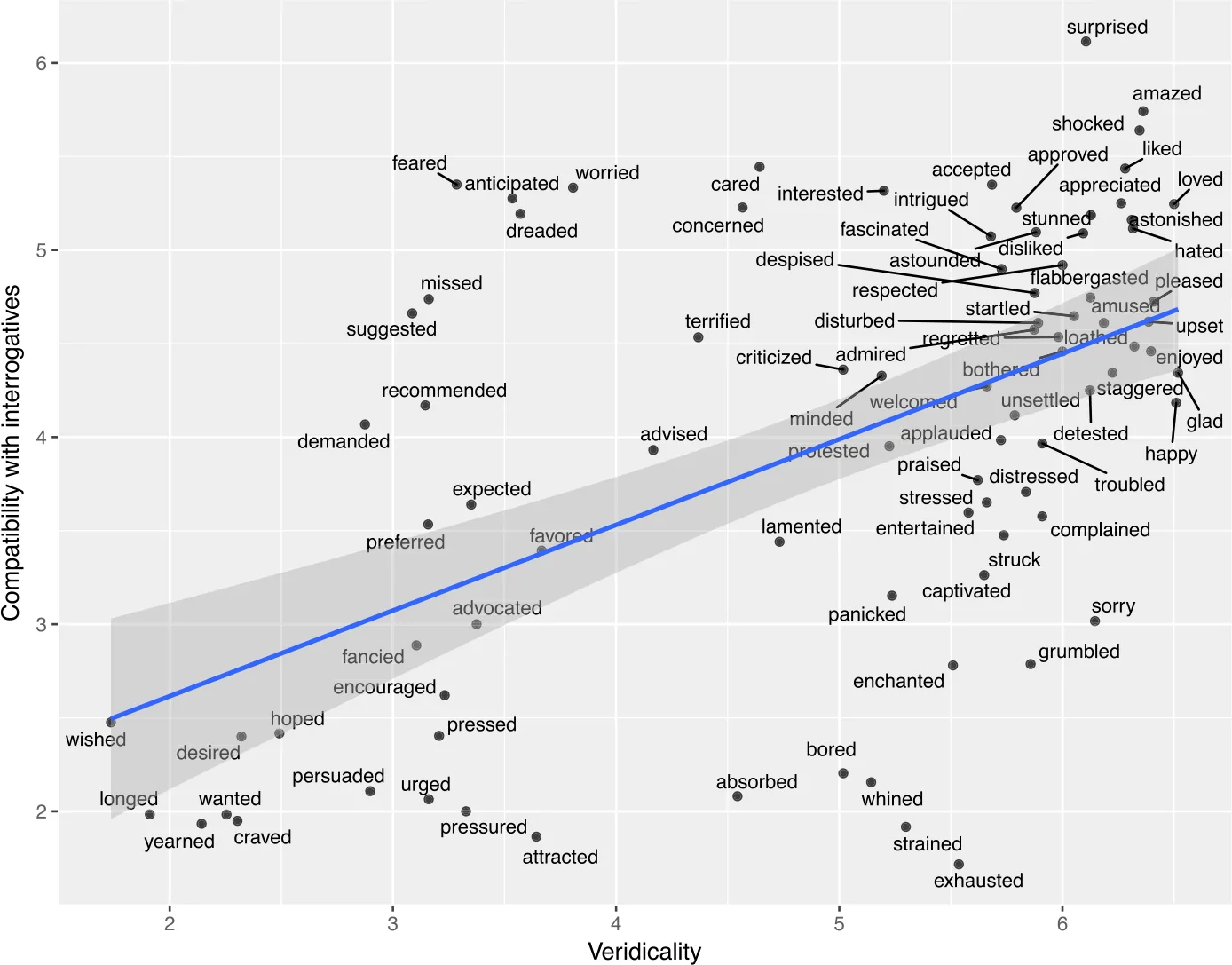
The plot of 87 preferential predicates in the experimental results by Uegaki (2022). The x-axis is the mean veridicality rating while the y-axis is the mean acceptability rating with interrogative complements. Mixed-effects logistic regression shows significant effect of Veridicality on Compatibility with interrogatives (p = 0.00154)

The results of the experiment are plotted in Fig. 1. Even though there is a correlation between veridicality and compatibility with interrogative clauses, predicates in the upper left quadrant constitute potential counterexamples to the generalization, as these are predicates that are judged to be compatible with interrogative complements while having low veridicality scores. For instance, while *fear* and *worry* received low veridicality scores as expected, they had relatively high compatibility with interrogative clauses. Stimuli sentences from the experiment exemplifying these judgments are shown below:^4^

(16)**Figure:**
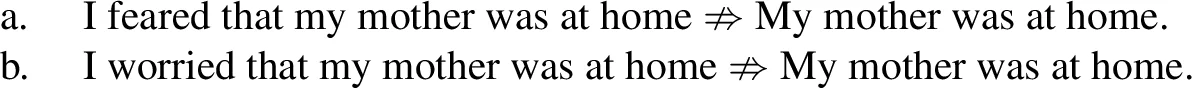

(17)**Figure:**
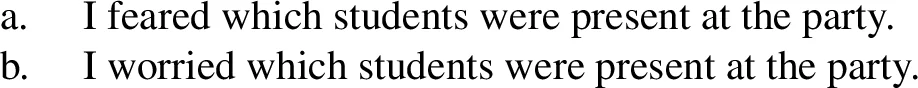

In addition, White (2021) reports several naturally occurring counterexamples where *fear* takes *whether*-complements, which we will discuss in Sect. 3.2.^5^

### Taking stock

U&S propose a descriptive generalization stating that all nonveridical preferential predicates are antirogative and provide a formal derivation for the generalization in terms of semantic triviality that results from combining nonveridical preferentials and interrogative clauses. Although a prediction of the generalization—that there is a correlation between veridicality and compatibility with interrogative clauses among preferential predicates—is experimentally supported by Uegaki (2022), the generalization also faces empirical challenges, as White (2021) and Uegaki (2022) show that there are examples of nonveridical preferential predicates taking interrogative clauses.

Thus, the descriptive generalization as proposed by U&S does not seem to stand, at least in its original form. At this point, we are left with two choices: (i) to abandon U&S’s project altogether, or (ii) to re-examine the original formal analysis by U&S, identify its crucial assumptions and qualify its actual empirical predictions. Although White (2021) proposes to pick the former option, we believe that it is worthwhile to explore the latter. Specifically, as noted earlier, U&S’s formal derivation of the incompatibility between a nonveridical predicate *V* and interrogative complements relies on two critical assumptions (18).

(18)**Figure:**
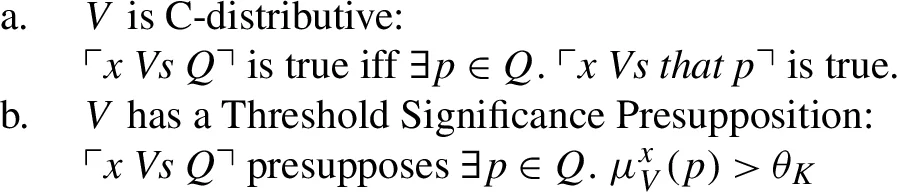

When both assumptions are met, *V* taking a question would result in a trivial meaning, which accounts for its ungrammaticality. However, if we remove either assumption, *V* taking a question would not result in a trivial meaning. Therefore, strictly speaking, U&S’s formal derivation actually makes the following empirical predictions (19).

(19)**Figure:**
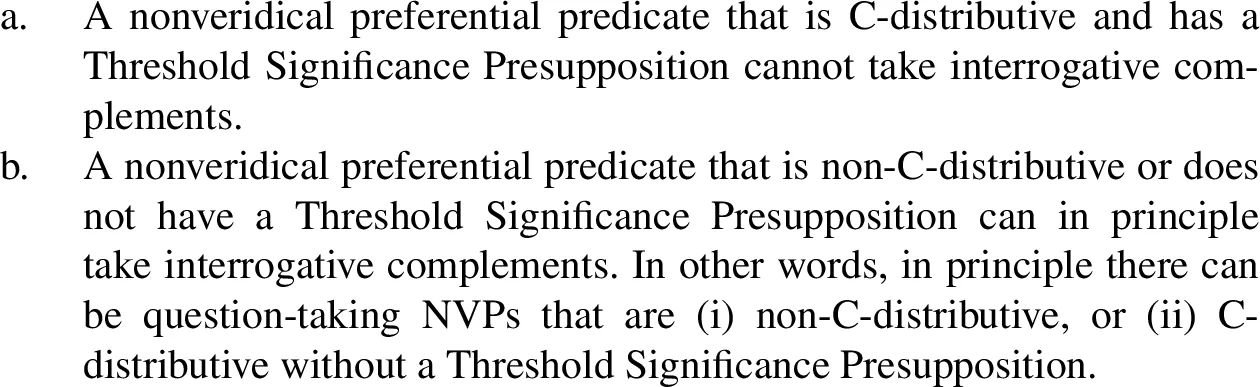

In what follows, we will provide empirical data supporting these predictions. Overall, we will show a more fine-grained classification of nonveridical preferential predicates than what U&S originally assumed, which is nevertheless anticipated by their formal analysis (even though they did not realize it).

## An updated classification of nonveridical preferentials

U&S made two assumptions about NVPs, namely, that they are C-distributive and that they trigger a Threshold Significance Presupposition. In this section, we show that neither assumption holds for all NVPs, and that some of the counterexamples to U&S’s original descriptive generalization can be categorized into two classes, based on whether the first or the second assumption does not hold for them. This leads to an updated classification of NVPs, which, to our knowledge, is novel. We also harness data from Mandarin Chinese, Japanese, Spanish, and Turkish to provide crosslinguistic support for this classification. These data were either attested online or constructed, and judged by at least two native speakers of the corresponding language.

### Non-C-distributive NVPs

Zooming out from the class of nonveridical preferential predicates for a moment, let us briefly review an example of a class of non-C-distributive predicates to get a sense of what such predicates might look like. Elliott et al. (2017) argue that *predicates of relevance* like *care* are non-C-distributive. Their argument goes as follows.

When *care* takes a declarative clause *p*, it entails and presupposes that the attitude holder believes *p* (at least under one of its uses, see below), as shown in (20).

(20)**Figure:**
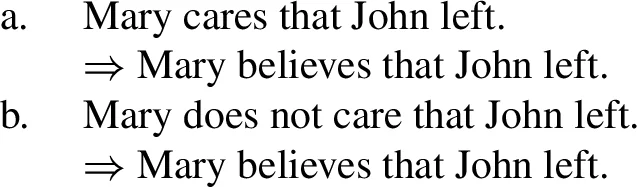

In contrast, when *care* takes an interrogative clause *Q*, as in (21-a), the entailment that the attitude holder believes an answer $p^{\prime }$ to *Q* disappears. To be clear, (21-a) is *compatible* with Mary believing an answer to the question and might even imply it, but if the predicate had been C-distributive, then this inference would have been an entailment. In particular, (21-b) would have been a contradiction: There, the second conjunct *wonder Q* entails that there is no answer to *Q* that the attitude holder believes. If the first conjunct *care Q* did entail that there was an answer $p^{\prime }$ to *Q* such that Mary cares that $p^{\prime }$, that would also entail that Mary believes $p^{\prime }$, in contradiction with the second conjunct. However, (21-b) is in fact not contradictory. Therefore, C-distributivity (18-a) does not hold for *care*.

(21)**Figure:**
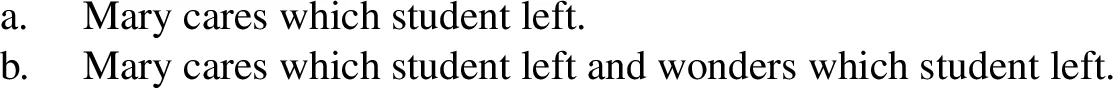

We note that Elliott et al.’s (2017) argument concerns the veridical/factive uses of *care*, and of predicates of relevance more generally, where this label comes from the observation that these predicates often presuppose the truth of their complement in addition to coming with the belief implication just seen. There is another use of *care* that neither implies truth nor belief (a nonemotive factive use of the predicate), which we discuss in the Appendix. This use can also be shown to be non-C-distributive, and the point that some responsive predicates are non-C-distributive stands regardless.

Given that not all predicates are C-distributive, there may in principle be NVPs that are non-C-distributive as well. When such predicates take interrogative clauses, their asserted content would no longer be predicted to be equivalent to the Threshold Significance Presupposition. Then, we would not expect the resulting meanings to be trivial. Therefore, we expect that there could be non-C-distributive NVPs that are compatible with interrogative clauses, and some attested examples in English and other languages are discussed below. For reasons that will become clear in Sect. 3.2, we already further divide such predicates by valence (i.e., whether they are evaluatively positive or negative).

#### Positive non-C-distributive NVPs

We first show that Mandarin Chinese *qidai* and Japanese *tanosimi-ni suru*, which can be roughly translated as *look forward to* in English, are evaluatively positive NVPs that are responsive and non-C-distributive.

Consider first the naturally occurring Mandarin sentences with *qidai* in (22) and (23), produced by a single speaker about an actor and his upcoming show. Here, and throughout, we indicate that an example comes from an online source with the superscript ^*γ*^ (Horn 2018). A link to the example is then provided in the Appendices.

When *qidai* takes an interrogative clause (22), there need not be an answer *p* to the embedded clause such that the attitude holder wants *p* to be true. Rather, the attitude holder simply wants to know what the true answer is.

(22)**Figure:**
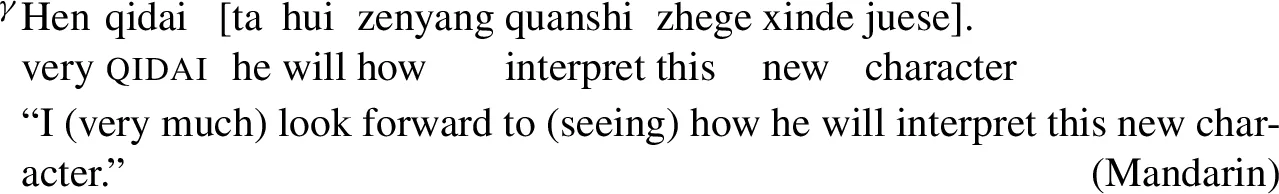

When *qidai* takes a declarative clause (23), it entails that the attitude holder wants the declarative clause to be true, but the declarative clause itself may or may not in fact be true, i.e., *qidai* is evaluatively positive but nonveridical.^6^

(23)**Figure:**
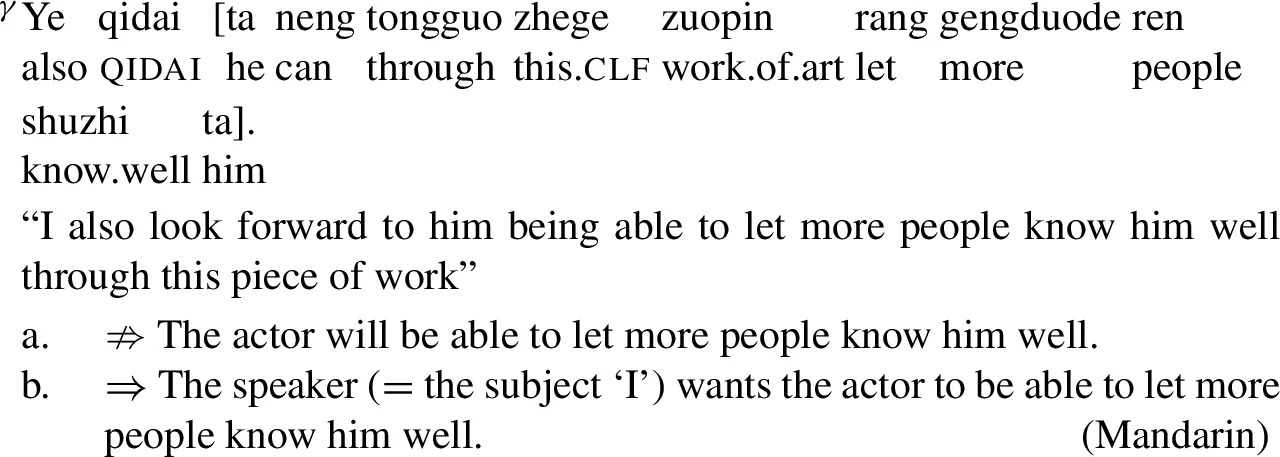

This means that *qidai(Q)* in (22) can be true without there being any answer *p* to *Q* such that *qidai(p)* is true (since *qidai(p)* entails that the attitude holder wants *p*). Therefore, *qidai* is non-C-distributive.

The same pattern holds for Japanese *tanosimi* ‘look forward to’.^7^ Sentences (24a) and (25)[a] are naturally occurring examples of the predicate taking questions and the resulting meanings are non-C-distributive, as they do not entail the corresponding (constructed) sentences in (24b) and (25)[b], regardless of the choice X of the person who wins Most Valuable Player (MVP) this year or the choice Y of the way in which Pattinson acts in The Batman.

(24)**Figure:**
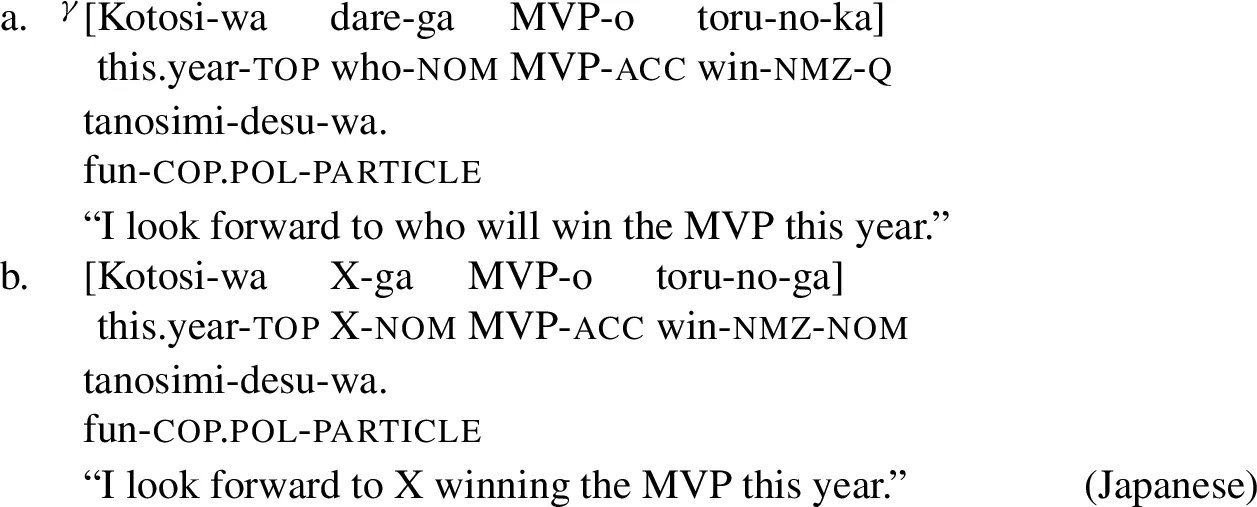

(25)**Figure:**
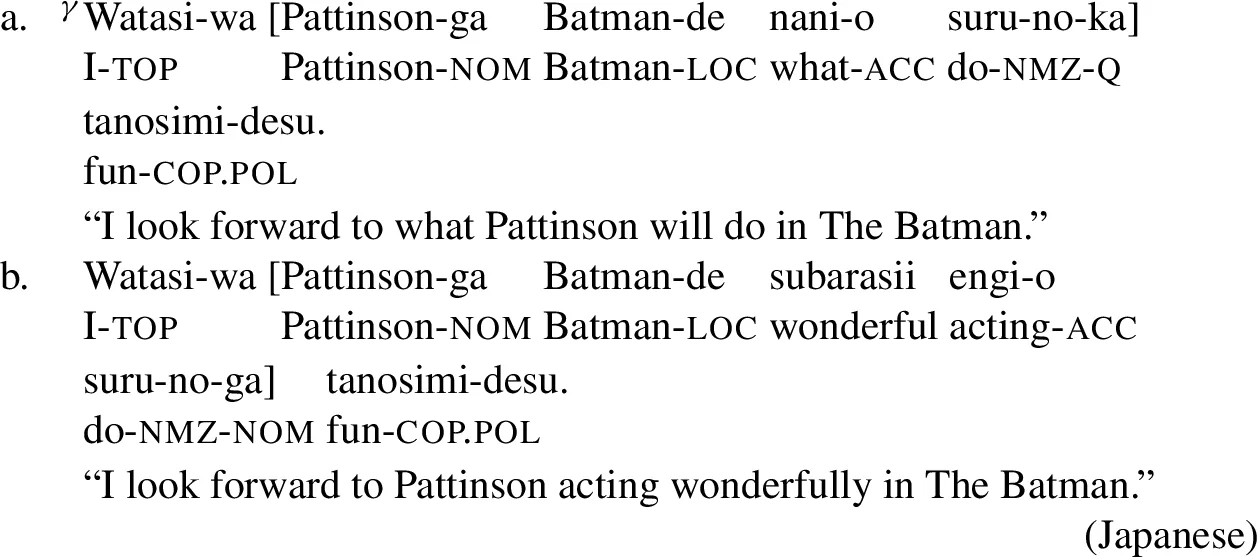

A natural question arises here about whether English *look forward to* also counts as an example of an evaluatively positive responsive NVP that is non-C-distributive. Here, we remain agnostic for now: We focus on clause-embedding predicates that take finite clauses and English *look forward to* does not take *that*-clauses, and it appears that not all speakers accept the predicate with nonfinite interrogative clauses either. Everybody seems to accept, for example, *look forward to* *seeing* *that/whether something is the case*. An analysis of such constructions would lead us astray of our main point, which is simply to say that positive non-C-distributive NVPs exist.

#### Negative non-C-distributive NVPs

Next, we suggest that there are also evaluatively *negative* NVPs that are responsive and non-C-distributive. Consider English *worry* with an experiencer subject. When it takes a declarative complement, the sentence is nonveridical (26-a). In addition, the predicate requires that the attitude holder must consider the embedded clause an undesirable possibility (26-b). That is, the predicate is evaluatively negative.

(26)**Figure:**
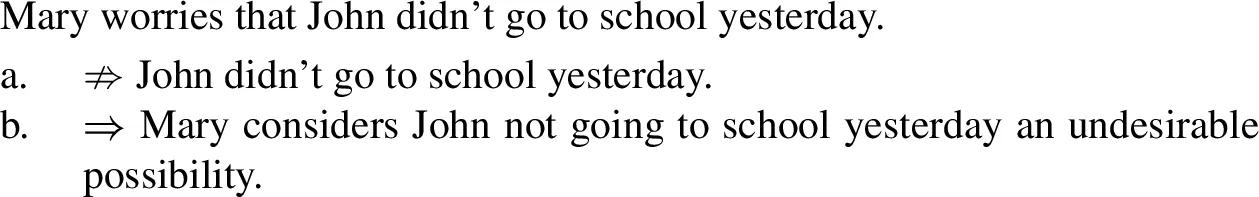

We can use the following context (27) to show that *worry* is non-C-distributive.

(27)**Figure:**
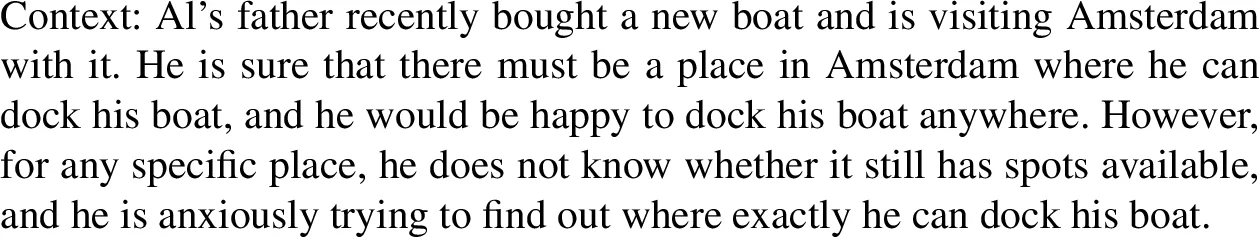

In this context, (28) is true.

(28)**Figure:**
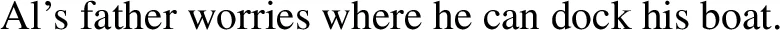

However, there is no particular place *Y* (for any choice of *Y*, e.g., Dock D) such that (29) is true. This is because (29) entails that Al’s father considers being able to dock the boat at *Y* an undesirable possibility, which is incompatible with the context in (27).

(29)**Figure:**
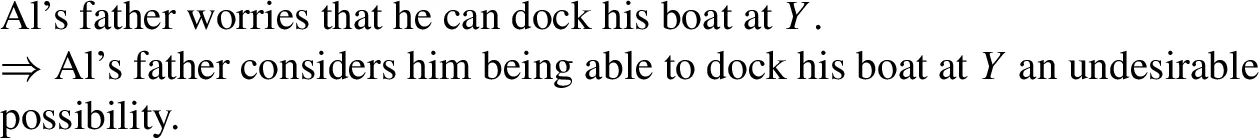

This means that *x worries Q* can be true without there being any answer *p* to *Q* such that *x worries that p* is true, and that the predicate is (or has a use that is) non-C-distributive.

About the predicate English *worry*, we will further note the following. First, when the predicate occurs in different syntactic frames (e.g., with a clausal or expletive subject, like in *That John didn’t go to school yesterday worries Mary* or *It worries Mary that John didn’t go to school*), or when the related deverbal adjectival construction with *be worried* is used, a veridical reading becomes available. We set this aside but refer the reader to Hartman (2012) for further discussion.

Second, when *worry* takes an interrogative clause, there might be a general preference for using the preposition *about* to introduce it. This might worry some readers about the possibility that, in the examples above, the predicate might not be composing directly with a clausal argument: The preposition might be playing a nontrivial role either in the syntax or the semantics. But, the naturally occurring examples in (30), where *worry* occurs preposition-less with interrogative clauses should serve to alleviate the worry. At least some English speakers do not require prepositions, and as we will suggest shortly, these are not required crosslinguistically.

(30)**Figure:**
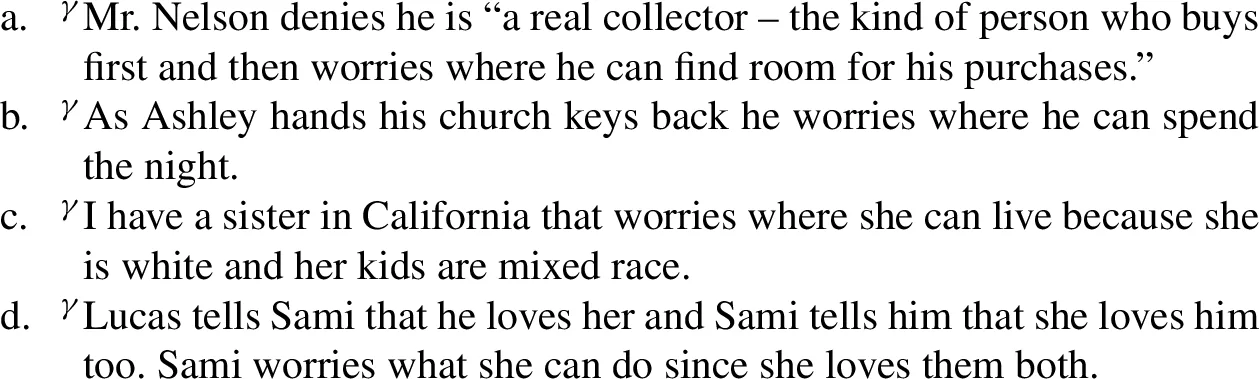

Crosslinguistically, Mandarin *danxin* (31), Japanese *sinpai-suru* (32), Spanish *preocupar* (33), Turkish *endişelen-* (34), which are close translations of English *worry*, all similarly exhibit non-C-distributivity: the (a)-sentences can be true without the (b)-sentences being true for any *Y*.^8^^,^^9^

(31)**Figure:**
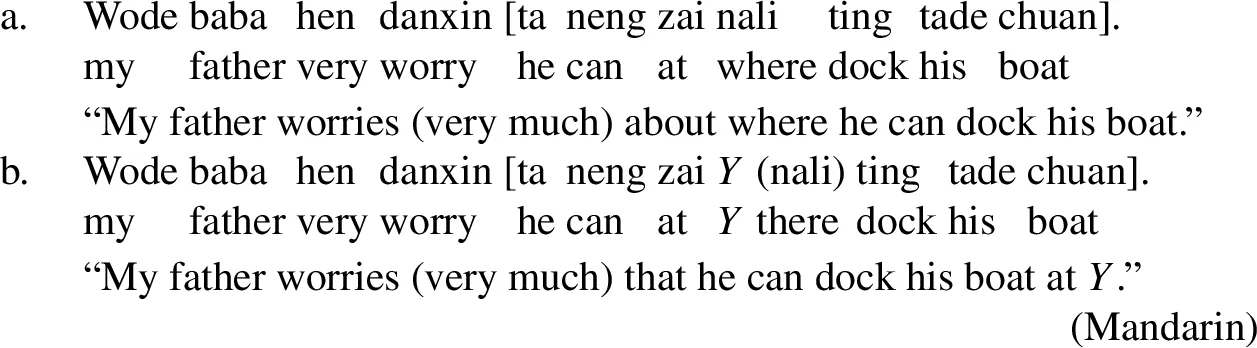

(32)**Figure:**
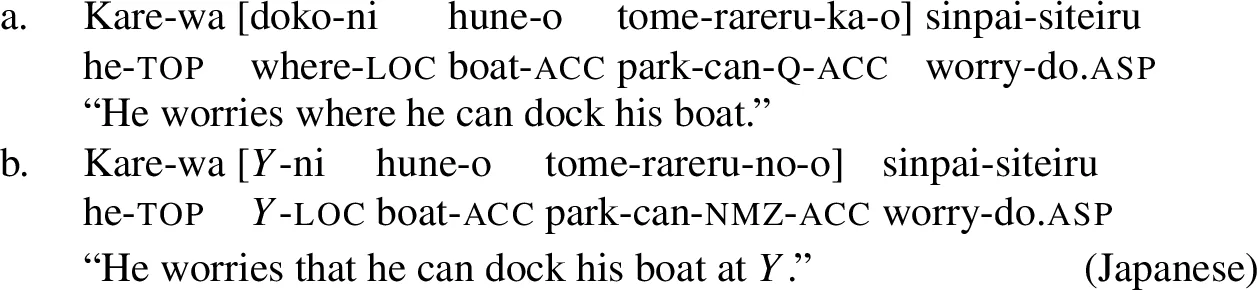

(33)**Figure:**
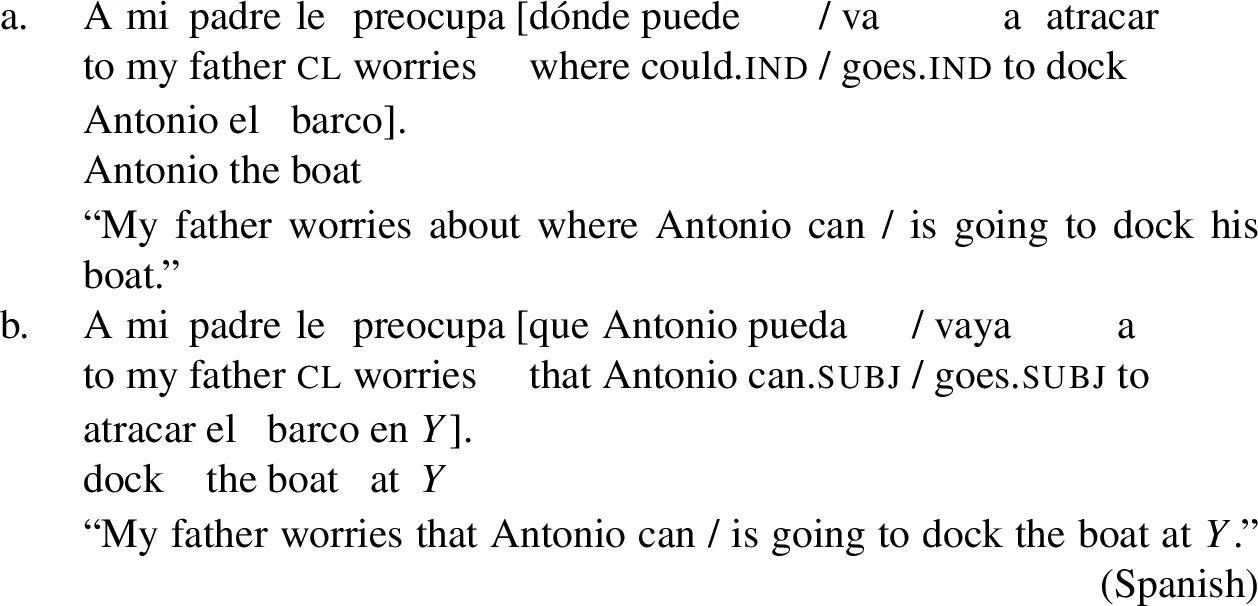

(34)**Figure:**
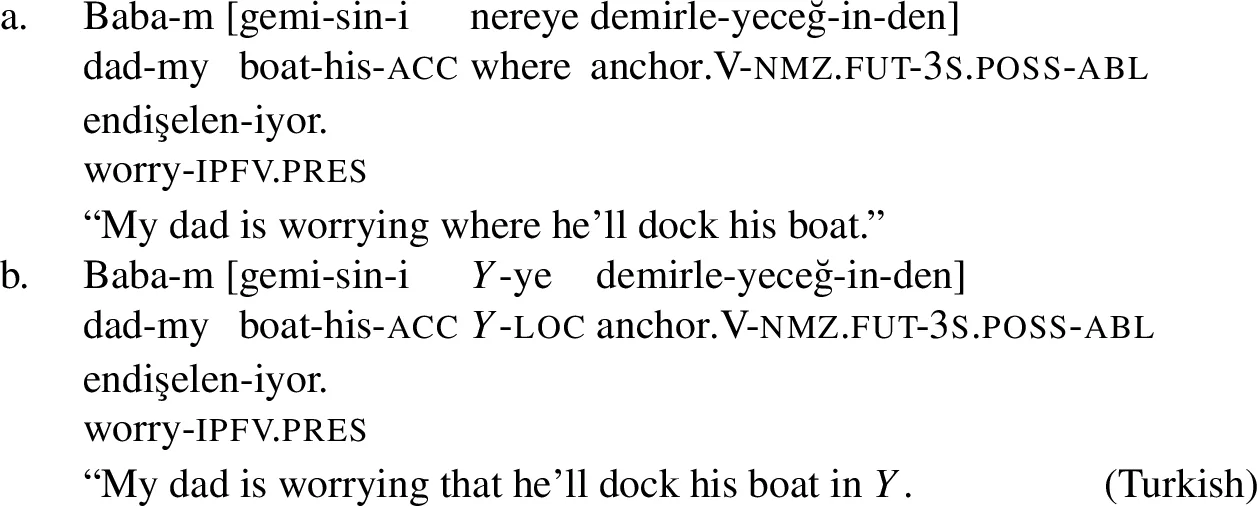

Other contexts, like the one in (35), can be imagined that also bring out natural non-C-distributive uses of such predicates.

(35)**Figure:**
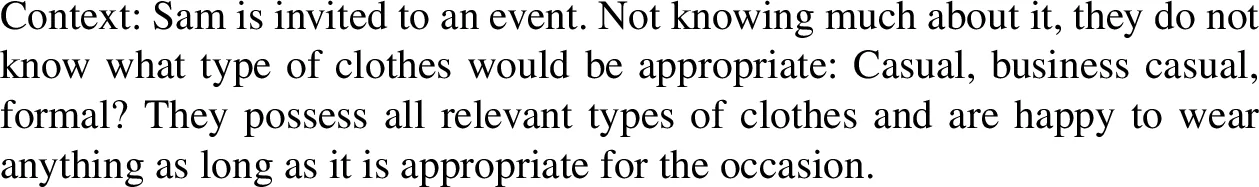

While (36a) can be true in this context, given that Sam is happy to wear anything, (36b) is false.

(36)**Figure:**
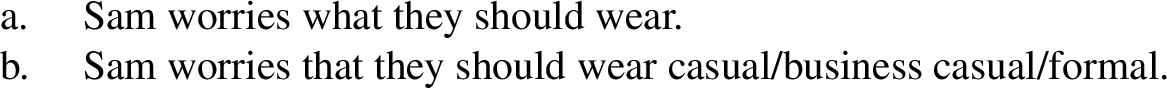

The examples below suggest the same conclusion in Mandarin, Japanese, Spanish, and Turkish.

(37)**Figure:**
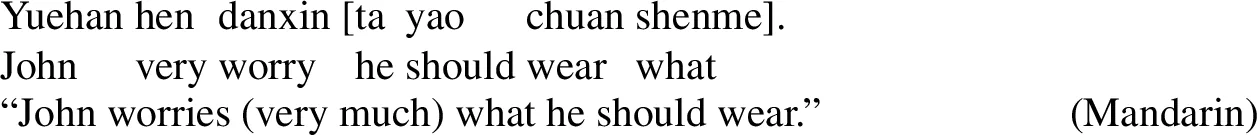

(38)**Figure:**
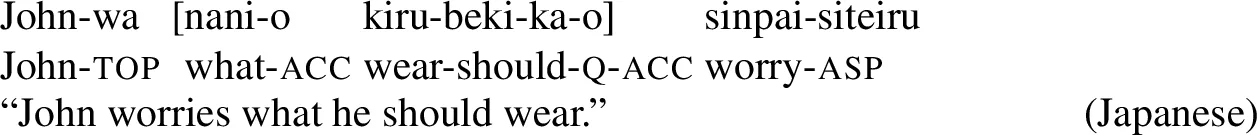

(39)**Figure:**
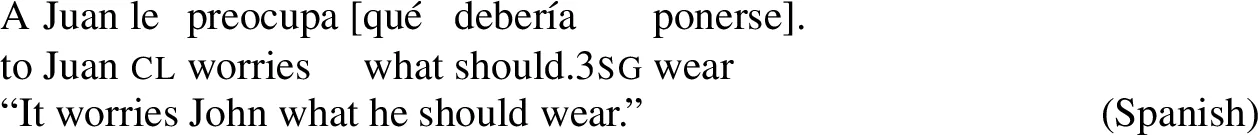

(40)**Figure:**
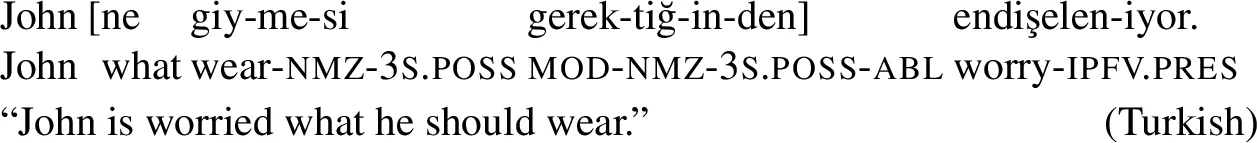

### Negative NVPs do not trigger the threshold significance presupposition

Our second class of question-embedding NVPs consists of predicates such as *fear*, which are *evaluatively negative* and C-distributive. Recall that in U&S’s formal derivation of the incompatibility between NVPs and embedded questions, the second crucial assumption is that NVPs have a Threshold Significance Presupposition, whose definition (18-b) is repeated below in (41).

(41)**Figure:**
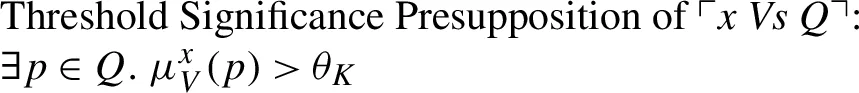

As discussed earlier, U&S’s empirical motivation for the Threshold Significance Presupposition is based on *evaluatively positive* predicates such as *like* and *be happy*, e.g., (42).

(42)**Figure:**
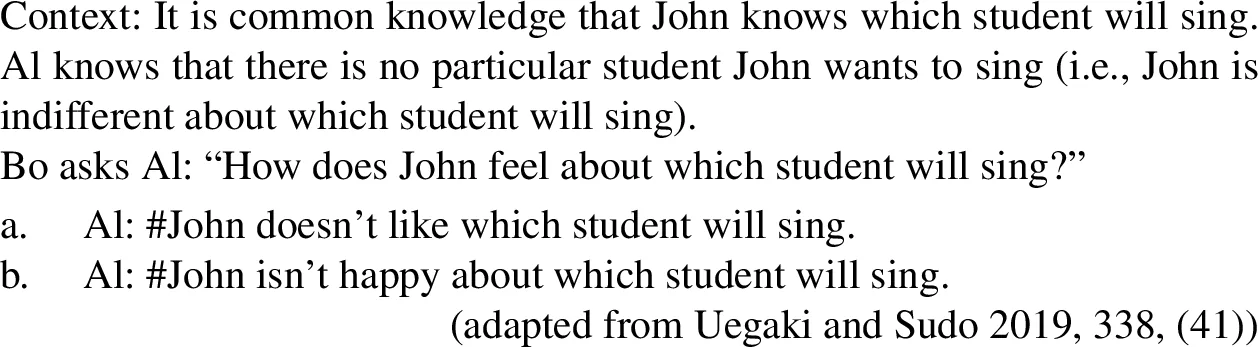

If there were no Threshold Significance Presupposition, Al’s answers in (42) would simply mean that it is not the case that John likes/is happy about which student will sing, and should be true in this context. However, empirically, such answers seem to presuppose the existence of a student John prefers to sing and, consequently, they feel infelicitous in the context where John does not have any preference. This point is also illustrated by (43).

(43)**Figure:**
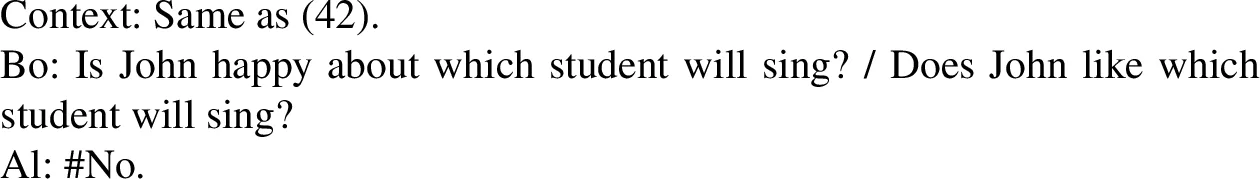

However, for evaluatively negative predicates such as *hate* and *be upset*, Al’s answers in (44) and (45) seem fine in the corresponding context where the Threshold Significance Presupposition is not met.

(44)**Figure:**
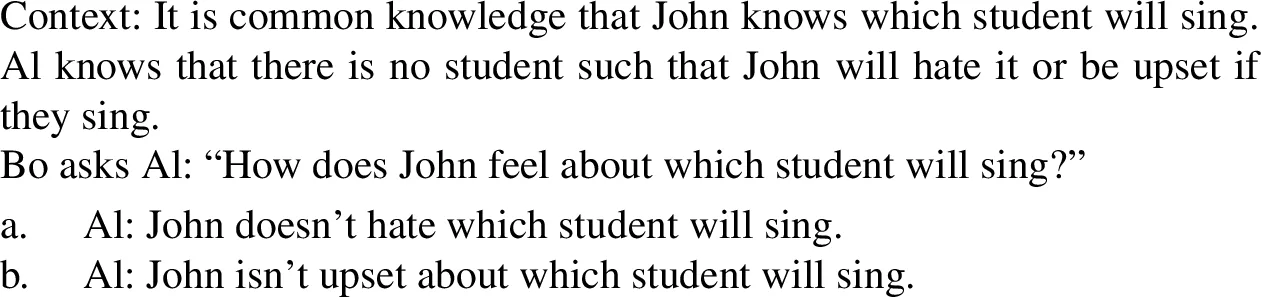

(45)**Figure:**
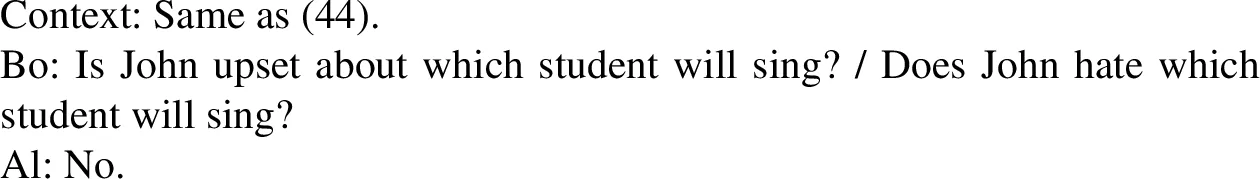

We take the contrast between evaluatively positive and negative predicates to suggest that evaluatively negative predicates in fact do not have a Threshold Significance Presupposition (see Klochowicz 2022 for the same conclusion; see also note 2).

Without this presupposition, U&S’s formal analysis predicts that for an evaluatively negative NVP such as *fear*, *A fears Q* simply means *for some p in Q, A fears that p*, which is not a trivial meaning. Therefore, we no longer expect negative NVPs to be incompatible with embedded questions.

This prediction of the revised U&S’s analysis is borne out. Regarding polar interrogative complements, White (2021) provides the following corpus examples (46).

(46)**Figure:**
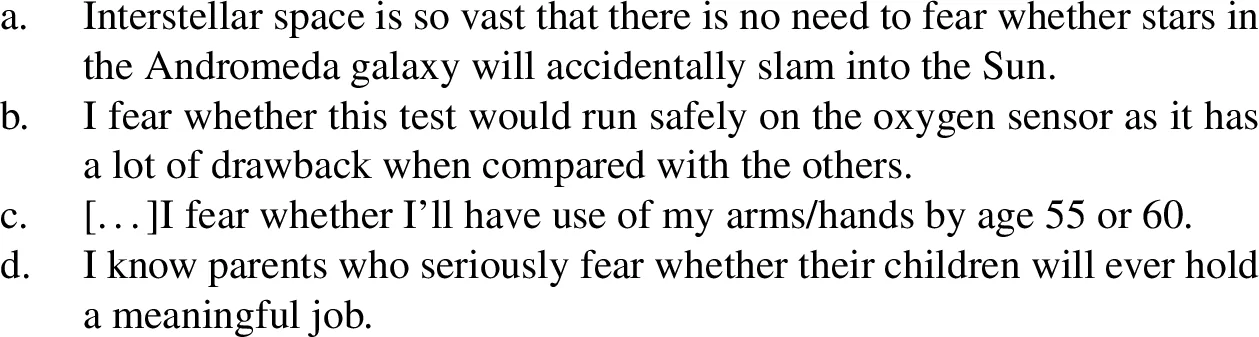

We can see that *fear whether p* is compatible with a scenario where the attitude holder fears that *p*, e.g., (46-a), as well as scenarios where the attitude holder fears that ¬*p*, e.g., (46-b)–(46-d).

And below are some naturally occurring examples of *fear* taking consistuent questions (47).

(47)**Figure:**
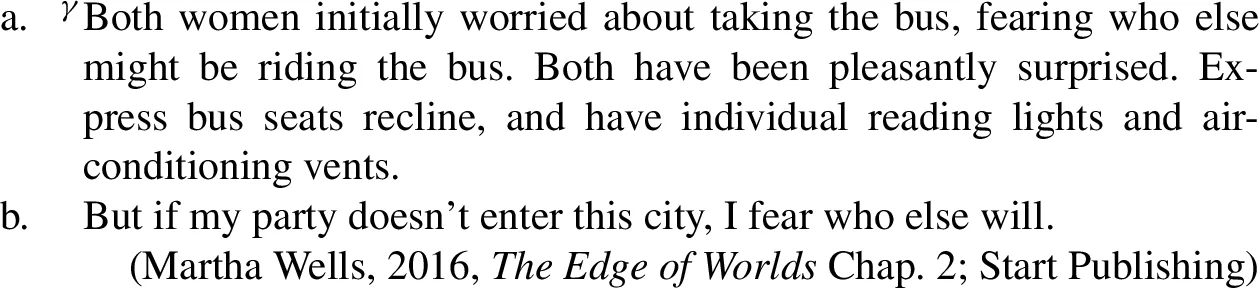

We can verify that *fear* indeed does not have a Threshold Significance Presupposition when it takes questions. For instance, (48) is compatible with a scenario where there exists no person *x* such that the women fear that *x* might be riding the bus. Contrast this case with negated *be happy* and *like* in (42).

(48)**Figure:**
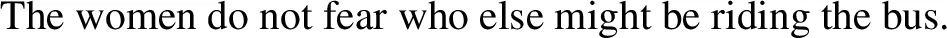

Crosslinguistically, there are further examples of evaluatively negative NVPs that can embed questions. For example, consider Turkish *kork-* “fear” (49).

(49)**Figure:**
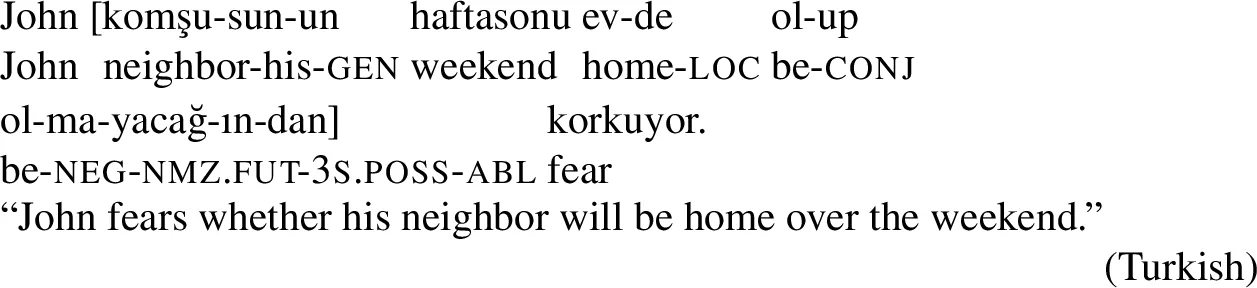

This sentence is felicitous in the GoodFriend context (50), where John fears that his neighbor will not be home, as well as the NoiseHater context (51), where John fears that his neighbor will be home.

(50)**Figure:**
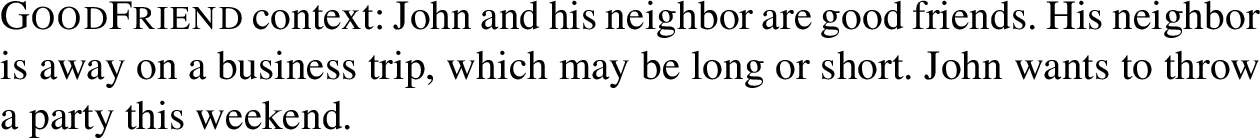

(51)**Figure:**
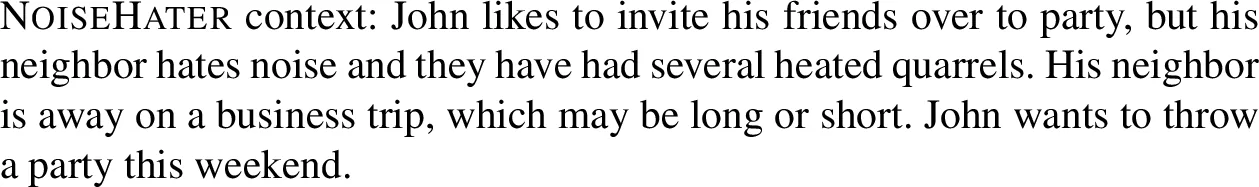

The predicate is also compatible with constituent questions (52).

(52)**Figure:**
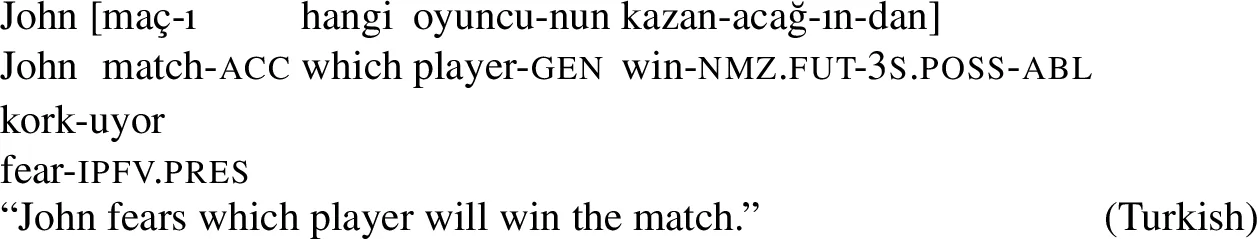

We can verify that it does not have a Threshold Significance Presupposition: (53) does not presuppose that there exists a player *x* such that John fears that *x* will win the match.

(53)**Figure:**
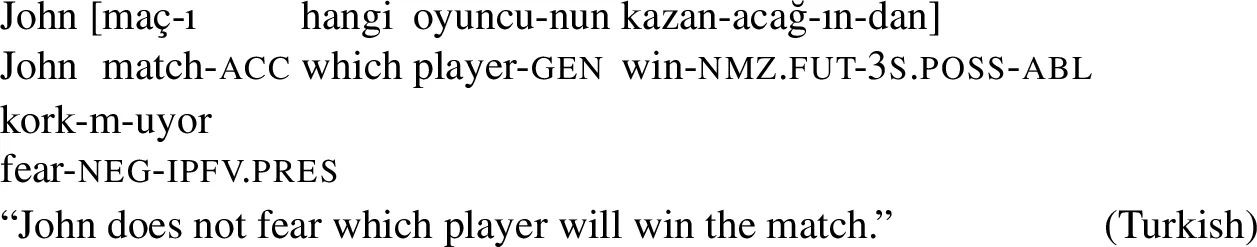

The same patterns hold for Japanese *osore(ru)* “fear.”

(54)**Figure:**
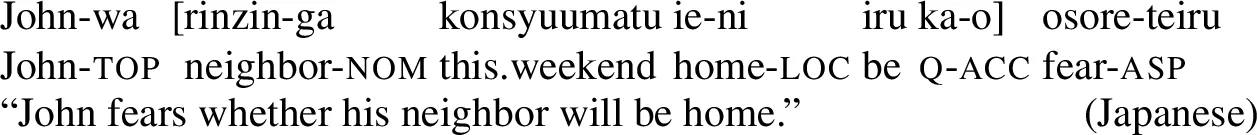

The sentence is felicitous in the GoodFriend context as well as the NoiseHater context. And the predicate is also compatible with constituent questions (55).

(55)**Figure:**
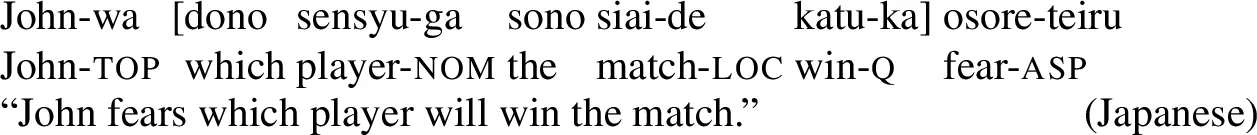

We can verify that it does not have a Threshold Significance Presupposition: (56) does not presuppose that there exists a player *x* such that John fears that *x* will win the match.

(56)**Figure:**
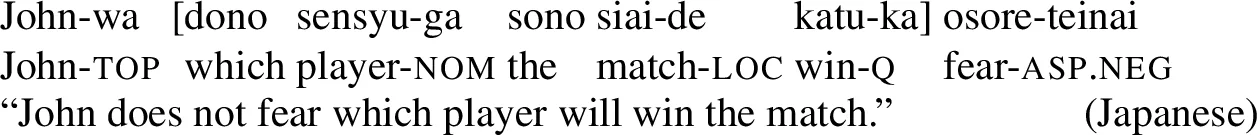

We emphasize that dropping the Threshold Significance Presupposition for evaluatively negative NVPs does not predict that they will always be compatible with interrogative clauses. It only says that, if they were to take interrogative clauses, the resulting meanings would not be trivial. However, there can be various other factors in a language that make such predicates incompatible with some or perhaps even all interrogative clauses.

For instance, while Spanish *temer* “fear” is compatible with constituent questions (57), it sounds quite bad with polar questions (58).

(57)**Figure:**
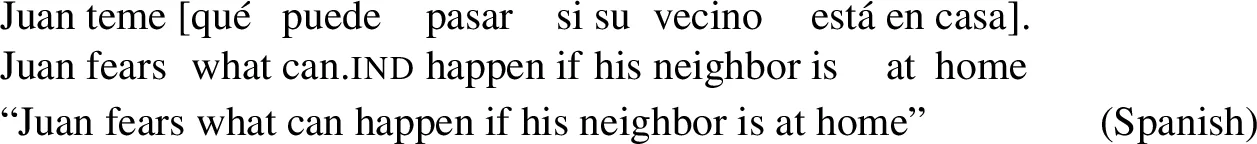

(58)**Figure:**
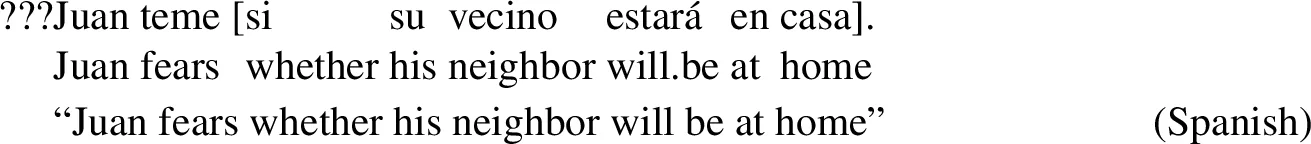

Mandarin *haipa* “fear” exhibits the opposite pattern. It is compatible with polar questions just like its English, Turkish, and Japanese counterparts (59).

(59)**Figure:**
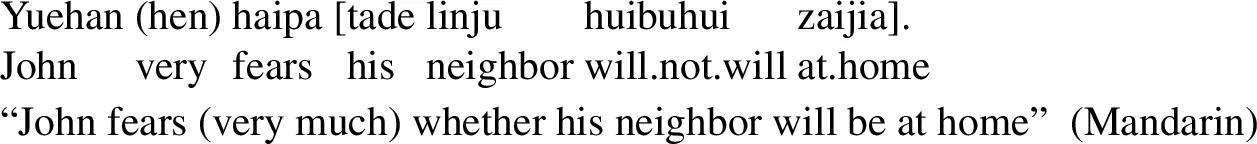

In contrast, *haipa* cannot take a constituent question. The only available interpretation of (60) is one where the wh-phrase is interpreted as an indefinite. In this case, the embedded clause is a declarative clause rather than a constituent question.^10^

(60)**Figure:**
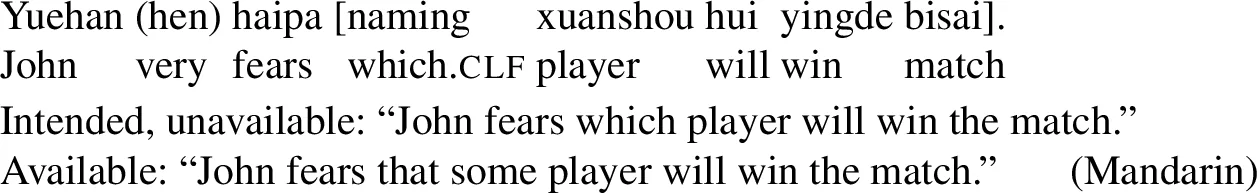

Explaining why the Spanish and Mandarin counterparts of *fear* are incompatible with some types of interrogative clauses is beyond the scope of this paper.^11^ For our purposes here, the fact that these predicates are compatible with *some* interrogative clauses provide further evidence for our proposal, according to which evaluatively negative predicates taking interrogative clauses will not result in semantic triviality.

### Summary of the updated classification of NVPs

In this section, we identified two relevant factors influencing a NVP’s compatibility with embedded questions: (i) whether the predicate is C-distributive, and (ii) the valence of the predicate (which correlates with whether the predicate has a Threshold Significance Presupposition). This leads to an updated classification of NVPs, summarized in Table 2, which contains two classes of responsive NVPs in addition to the class of antirogative NVPs predicted by Uegaki and Sudo (2019).

**Table 2:** Summary of our updated classification of NVPs

Class	Lexical semantics		Combinatorial	Examples
	C-distr.	Valence	Classical composition with *Q*	
1	no	positive	✓	Mandarin *qidai*, Japanese *tanosimi*
	no	negative	✓	English *worry*, Japanese *sinpai-suru*,
				Mandarin *danxin*, Spanish *preocupar*,
				Turkish *endişelen-*
2	yes	negative	✓	English *fear*, Japanese *osore(ru)*,
				Turkish *kork-*
3	yes/NA	positive	*	English *hope*, Japanese *nozomu*,
				Mandarin *xiwang*, Spanish *esperar*,
				Turkish *um-*

First, for NVPs that are non-C-distributive, regardless of whether they have a Threshold Significance Presupposition when they take questions, the asserted content is not equivalent to it and hence no triviality arises. Therefore, U&S’s formal analysis does not rule out such predicates, and we expect to find non-C-distributive predicates that can take embedded questions. Examples of evaluatively positive question-taking NVPs that are non-C-distributive include Mandarin *qidai* and Japanese *tanosimi*, which can be roughly translated as English *look forward to*. Examples of evaluatively negative responsive NVPs that are non-C-distributive include English *worry* (with an experiencer subject) and its close translations in Japanese (*sinpai-suru*), Mandarin (*danxin*), Spanish (*preocupar*), and Turkish (*endişelen-*).

Second, for NVPs that are evaluatively negative, we showed that they do not have a Threshold Significance Presupposition. As a result, such predicates taking interrogative clauses would not lead to trivial meanings. Therefore, U&S’s formal analysis does not rule out such predicates, and we expect to find a class of question-taking NVPs that are evaluatively negative and C-distributive. Examples of such predicates include English *fear* and its close translations in Japanese (*osore(ru)*) and Turkish (*kork-*).

Finally, for positive NVPs that are C-distributive, U&S’s formal analysis predicts that they are incompatible with embedded questions due to the asserted content being equivalent to the Threshold Significance Presupposition.^12^ We maintain their analysis for this class of predicates, which includes English *hope* and its close translations in other languages. As a reminder, our classification here only concerns cases of what we call classical composition with questions, where the predicate takes the symmetric denotation of its embedded question as its semantic argument.

## General discussion and conclusion

In describing the distribution and the interpretation of embedded interrogatives with different preferential predicates, we find support for the view that the restrictions and freedom in the combinatorial properties of clause-embedding predicates are to a large extent governed by their semantic properties. While this is a point of consensus in the semantic literature on clause-embedding, disagreements remain about which semantic properties are relevant (veridicality? preferentiality? neg-raising? eventivity? and so on), and about which generalizations are empirically motivated that relate clause-embedding predicates’ semantic and combinatorial properties.

One point of tension is between Uegaki and Sudo (2019), who suggest that all nonveridical preferential predicates have to be antirogative, and White (2021), who produces examples where *fear*, a paradigmatic nonveridical preferential predicate, *is* attested with embedded questions. This suggests that the original empirical generalization is false and leads to the natural conclusion that its explanation might be misguided, and that we should look for properties other than (non-)veridicality and preferentiality to explain the combinatorial properties of attitude predicates.

In this paper, we take this challenge seriously. In addition to spelling out the interpretive properties of *hope* and *fear* when they combine with embedded questions, we expand the repertoire of counterexamples to Uegaki and Sudo’s (2019) empirical generalization and check the behavior of these predicates’ counterparts in Japanese, Mandarin, Spanish, and Turkish. Concretely, we propose an updated classification of NVPs based on two lexical semantic properties: C(lausal)-distributivity and valence, that is, whether the equivalence holds that ⌜*x* *Vs* *Q*⌝ iff there is an answer *p* to *Q* such that ⌜*x* *Vs* *that* *p*⌝, and whether a preferential predicate *V* is evaluatively positive or negative. Predicates that are either non-C-distributive or negative are generally free to combine with embedded questions. We consider these to be genuine counterexamples to Uegaki and Sudo’s (2019) empirical generalization but point out that Uegaki and Sudo’s formal analysis in fact makes the right predictions about these predicates, because the relevant semantic properties that would lead to triviality when NVPs take questions in fact do not hold for such predicates. Among such responsive NVPs, the C-distributive ones, which are evaluatively negative, describe a relation between individuals and any possible answer to an interrogative (i.e., *fear whether p* may either be paraphrased as *fear that p* or *fear that not p*), and the non-C-distributive ones, a relation between individuals and questions that is not reducible to relation to any particular answer. The remaining class of NVPs, i.e., those that are both positive and C-distributive such as *hope*, are difficult to combine with embedded questions. This leads us to maintain Uegaki and Sudo’s (2019) original proposal for these predicates, which appeals to the Threshold Significance Presupposition to derive that they should be antirogative (see Sect. 2.1).

To corroborate or call into question our current findings, it is crucial to expand our search space to more predicates, and to languages different from the ones surveyed here. If our findings here are on the right track, the prediction is that predicates that trigger the Threshold Significance Presupposition and that are C-distributive will be restricted in their ability to combine with embedded questions—in line with Uegaki and Sudo’s (2019) original observation. (We have only found evaluatively positive predicates with the Threshold Significance Presupposition, but do not exclude the possibility that there might be negative ones as well.) In contrast, predicates that lack either one of these properties are expected to be freer in their combinatorial properties.^13^

## Appendix A: Nonveridical *care*

Elliott et al.’s (2017) argument shows that *emotive factive* uses of *care* are non-C-distributive. We further suggest that English *care* also has uses that are not emotive factive. For simplicity, we will call such uses *nonveridical*. Consider the following naturally occurring example where a law firm partner talks about qualities they look for.

Here, *care* is nonveridical in *I care that you will work hard*: It does not entail that the addressee will work hard or that the attitude holder (which is the speaker in this case) believes that the addressee will work hard.^14^ The attitude holder merely wants it to be the case that the addressee will work hard.

One crucial difference between veridical and nonveridical uses of *care* is whether the attitude holder must have a preference for the embedded clause to be true. For veridical uses of *care*, it is possible that the attitude holder does not prefer that the embedded clause be true (62).

In contrast, we observe that nonveridical uses of *care* entail that the attitude holder wants the declarative complement to be true (63). For instance, in a nonveridical context (64), given that Al (not knowing whether Bo left) wants it to be the case that Bo left, (64-a) is judged acceptable. This is because (64-a) is true under a nonveridical use of *care*. In contrast, (64-b) is not acceptable in this context, because it is not true under the nonveridical use.

Based on the observation that nonveridical uses of *care* entail the attitude holder wants the declarative complement to be true, we can show such uses are also non-C-distributive: (65) can be true without there being a student *x* such that Al wants it to be the case that *x* left. For instance, (65) is true in context (66), whereas (67) is not true in this context for any student *x*.

The discussion above suggests that nonveridical uses of *care* are evaluatively positive, and if a question-taking *care*, such as the one in (65), could have a nonveridical use, it would be non-C-distributive. However, given that in most cases the salient reading of *care* is the veridical one, one may still question whether the same *care* is involved in its nonveridical uses and when it takes questions in (65). Therefore, we only take nonveridical uses of *care* as a tentative example of an evaluatively positive non-C-distributive NVP.

## Appendix B: References to naturally occurring examples

• Exx. (22) & (23-b): https://www.ximalaya.com/ask/q8788545?source=m_jump

• Ex. (24-a): https://www.smbc.co.jp/sponsorship/smbcnipponseries/column/article01/

• Ex. (25-a): https://www.marvelvsdc.xyz/entry/dcfu_89

• Ex. (30-a): https://www.nytimes.com/1972/09/08/archives/painted-furniture-new-interest-in-an-old-art.html

• Ex. (30-b): https://www.thefreelibrary.com/Our+pick+of+the+best+soaps+%26telly.-a0295046793

• Ex. (30-c): https://www.city-data.com/forum/las-vegas/340170-rainbow-family-moving-vegas-30-days-2.html

• Ex. (30-d): https://tvmegasite.net/updates/days/2004/days-12-10-04.shtml

• Ex. (47-a): https://eu.heraldtribune.com/story/news/2012/11/11/on-scats-express-bus-its-life-in-the-fast-lane/29134294007/

• Ex. (61): https://www.lawpracticetoday.org/article/right-kind-grit-succeed/

Except for (30-b), all the pages have been archived using Internet Archive’s Wayback Machine (https://web.archive.org/).

## Footnotes

1 Following Dayal (2016), in this paper, we use the terms *interrogative (clause)* and *question denotation* to unambiguously refer to the respective syntactic and semantic notions, and the term *question* as a cover term for both notions. This will allow us to use more natural terms in some cases, e.g., *alternative question* instead of *alternative interrogative* when talking about the syntactic object.

2 We presented this analysis at the Generative Linguistics in the Old World (GLOW) conference in April 2022. Parts of a similar analysis were simultaneously and independently developed by Klochowicz (2022).

3 A reviewer notes that positing a Threshold Significance Presupposition is not the only way to account for the data point in (14), and by extension, for the antirogativity of NVPs. One could assume, they note, that *be happy* is neg-raising(-like) in its at-issue content, with *x isn’t happy that p* implying that *x* prefers not *p* to *p*. We agree that there are interesting alternatives to assuming the Threshold Significance Presupposition, but our more modest goal here is to present U&S’s account and clarify its predictions, and hence the discussion of alternatives is beyond the scope of this paper.

4 The actual prompts in Uegaki (2022) were as follows, where *V* is the predicate in question:

5 White (2021) also reports naturally occurring counterexamples where *hope* takes *whether*-complements. However, the grammaticality status of these examples is contested: some speakers find such examples completely acceptable, some find them completely unacceptable, and others find them marked to various degrees. Therefore, following the advice from a reviewer and the editor, we leave these examples out in this paper. Additionally, we believe such examples need to be analyzed using nonclassical composition, which falls outside the scope of U&S’s original paper.

6 A reviewer points out that from a morphological and etymological perspective, since *qi* means to expect (in the epistemic sense) and *dai* means to wait, one might question whether *qidai* really entails preference instead of having it only as a pragmatic inference. We confirm that *qidai* indeed entails preference in its contemporary usage (at least in the varieties spoken by our consultants), as can be seen by the contrast between *qidai* and *yuqi*. Denying preference is coherent only after *yuqi*.

7 This predicate is unrelated to the Chinese predicate *qidai* above. Morphologically, *tanosimi* itself is a nominalized form of the verb *tanosimu*, meaning ‘to enjoy’. Combined with the copula *da* (or its polite counterpart *desu* as in (24) or with the light verb *suru*, it describes the state of positive anticipation.

8 Although some authors classify Spanish *preocupar* ‘worry’ as emotive factive (e.g., Villalta 2008), (33-b) crucially allows for a nonveridical interpretation of the *that*-clause. The difference in embedded mood between (33-a) and (33-b) is due to the fact that embedded interrogative clauses obligatorily appear in the indicative while *that*-clauses under *preocupar* appear in the subjunctive by default—though see Falkner (2022) for special discourse effects triggered by the indicative.

9 The Turkish translation (34) uses the future instead of a possibility modal in the embedded clause because (for reasons that we do not know) the latter sounds less natural. Despite this difference, our crucial point remains, i.e., the predicate is non-C-distributive because the (a) sentence can be true without the (b) sentence being true for any place *Y*.

10 We can further illustrate the unavailability of the embedded question interpretation by considering sentences that do not have a wh-indefinite interpretation. For instance, (60) is totally unacceptable.

11 The incompatibility might be due to purely syntactic constraints that are language-specific, and in principle we do not rule out the possibility that it might be due to subtle meaning differences between English *fear* (and its Turkish and Japanese counterparts) on the one hand and the Spanish and Mandarin counterparts on the other. However, so far we have not detected any meaning difference between these predicates from different languages that would tease them apart.

12 Given that such predicates cannot take interrogative clauses, they are only presumed to be C-distributive according to U&S’s original analysis based on the fact that most responsive predicates are C-distributive. This is why the C-distributivity of this class is marked as NA/yes in Table 2.

13 But there may well be additional syntactic and semantic factors that make them incompatible with questions. Also, it is important to keep in mind that one should not take for granted that a predicate *P* in one language and its intuitive counterpart $P^{\prime }$ in another language will share the relevant semantic properties discussed in this paper. For instance, if a language only has a non-C-distributive positive predicate similar to Mandarin *qidai* or Japanese *tanosimi* (i.e., it does not have a predicate similar to *hope*), speakers of that language may use such a predicate to translate *hope*. In this respect, semantic properties such as C-distributivity can help diagnose whether a predicate’s intuitive counterpart in a different language is really equivalent.
